# Use of Aflibercept to Treat Choroidal Neovascularization Secondary to Choroidal Osteoma in a 7‐Year‐Old Child: A Multimodal Imaging Case Report

**DOI:** 10.1155/crop/5548292

**Published:** 2026-08-10

**Authors:** Abdullah Altalhi, Anas Alqurashi, Mohammed Albassam, Abdulmalik Alyahya

**Affiliations:** ^1^ Division of Vitreoretinal Surgery, King Khaled Eye Specialist Hospital & Research Center, Riyadh, Saudi Arabia; ^2^ Research Department, King Khaled Eye Specialist Hospital & Research Center, Riyadh, Saudi Arabia; ^3^ Department of Ophthalmology, Makkah Health Cluster, Makkah, Saudi Arabia

**Keywords:** aflibercept, anti–vascular endothelial growth factor therapy, choroidal neovascularization, choroidal osteoma, pediatric retinal disease

## Abstract

**Purpose:**

This study is aimed at reporting a case of choroidal osteoma in a young boy complicated by choroidal neovascularization (CNV) and describing its clinical course over 9 months following intravitreal aflibercept injections.

**Observation:**

A 7‐year‐old boy presented with a 2‐month history of decreased vision in his left eye. Clinical examination and multimodal imaging revealed a central choroidal osteoma complicated by an active CNV with associated subretinal hemorrhage and fluid. The patient was treated with intravitreal aflibercept under general anesthesia. Follow‐up visits demonstrated early anatomical improvement, with resolution of subretinal hemorrhage and fluid. However, recurrent CNV activity and fluctuating visual acuity were observed.

**Conclusion:**

Although choroidal osteoma is more commonly reported in adult females, it should also be considered in young boys. Early treatment with aflibercept was associated with short‐term anatomical and transient functional improvements in the management of CNV secondary to choroidal osteoma. However, visual acuity fluctuated during follow‐up visits, necessitating recurrent CNV injections. Therefore, regular follow‐up is essential due to the risk of recurrence and to preserve long‐term visual acuity. Given the rarity of this condition, further studies with larger sample sizes and extended follow‐up periods are required to evaluate the effectiveness of anti–vascular endothelial growth factor therapy and establish evidence‐based management guidelines.

## 1. Introduction

Choroidal osteoma (CO) is a rare, benign ossifying tumor of the choroid characterized by the replacement of normal choroidal tissue with mature cancellous bone [1, 2]. It commonly originates in the peripapillary region and may extend to the macula, resulting in gradual visual field and acuity loss in later stages [2]. The pathogenesis remains unclear, although several hypotheses have been proposed, including a hormonal mechanism, which attempts to explain why females in their 20s are more affected than males [3]. Therapeutic options include surgical excision, laser therapies, and intravitreal anti–vascular endothelial growth factor (VEGF) injections when CO is complicated by choroidal neovascularization (CNV); prior reports have most commonly described the use of ranibizumab or bevacizumab [4–6]. The present report describes the case of a 7‐year‐old boy diagnosed with CO complicated by active CNV and treated with aflibercept (Eylea; Regeneron Pharmaceuticals, Tarrytown, New York, United States), an anti‐VEGF agent approved by the US Food and Drug Administration. Ethical approval was obtained from the institutional review board of the King Khaled Eye Specialist Hospital and Research Center (Riyadh, Saudi Arabia) (RP 25178‐CR), and written informed consent was obtained from the patient′s guardian for publication of this case report and accompanying images. Patient details have been sufficiently anonymized according to the ICMJE guidelines.

## 2. Case Presentation

**Figure 1:**
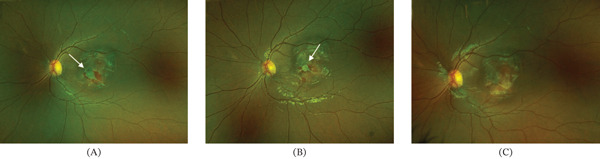
Ultrawide pseudocolor fundus images of the left eye across three consecutive visits. (A) At the first visit, a whitish subfoveal lesion measuring approximately half a disk diameter was observed, surrounded by a fresh subretinal hemorrhage (white arrow). (B) At the 1‐month follow‐up, the subretinal hemorrhage was reduced (white arrow). (C) At the 3‐month follow‐up, a complete resolution of the subretinal hemorrhage was observed.

**Figure 2:**
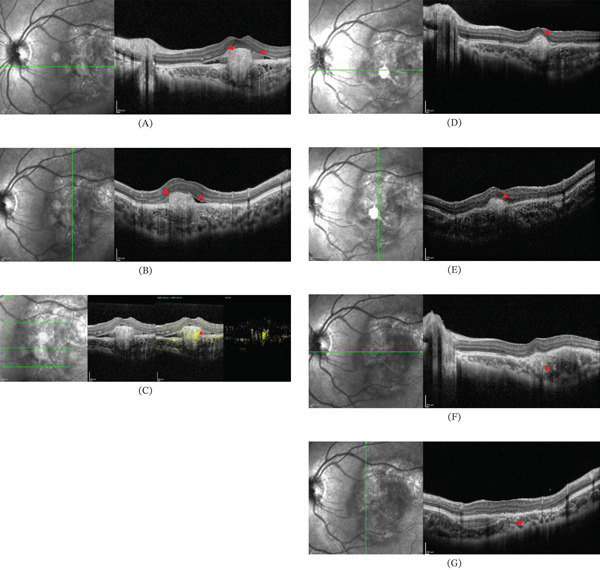
Left macular OCT and OCT‐A of the left eye across three consecutive visits. (A, B) At the first visit, structural OCT revealed subretinal fluid and hemorrhage (red arrows). (C) OCT‐A at the first visit demonstrated choroidal neovascularization (CNV) with active intralesional blood flow (red arrow). (D, E) At the 1‐month follow‐up, OCT revealed decreased subretinal fluid and hemorrhage (red arrow). (F, G) At 3 months of follow‐up, OCT revealed the complete resolution of subretinal fluid and blood. Hyperreflective dots and thickened choroidal areas were observed (red arrow).

**Figure 3:**
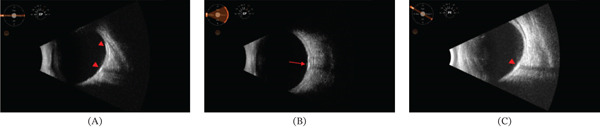
B‐scan ocular ultrasound of the left eye at the first visit. (A–C) Ocular wall calcification (red arrows) is shown with posterior acoustic shadowing involving the left macular region.

**Figure 4:**
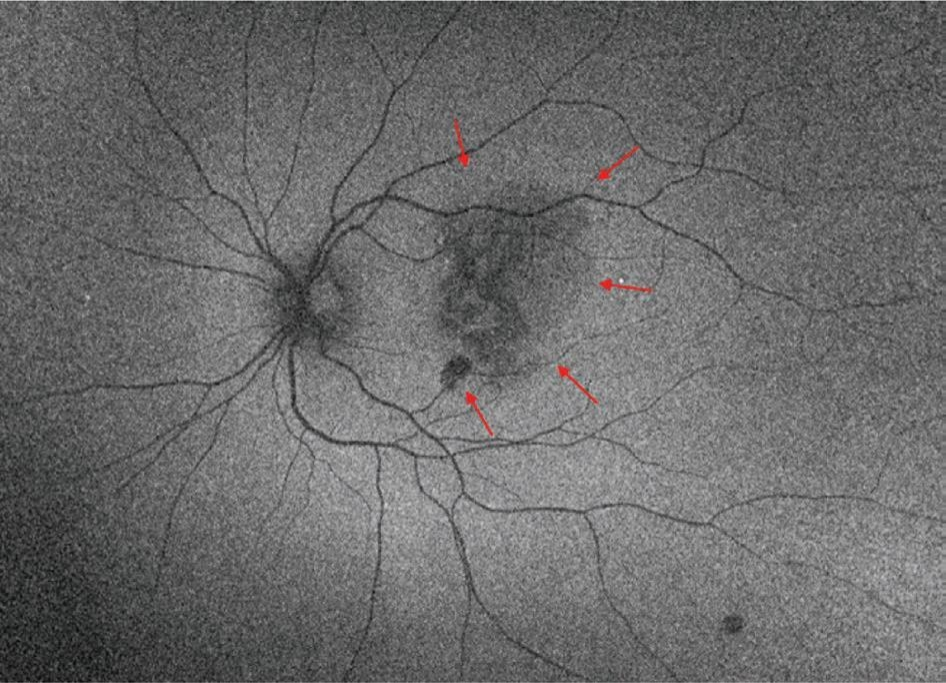
Fundus autofluorescence image of the left eye at the 1‐month follow‐up showing a large area of hypoautofluorescence corresponding to the resolving subretinal hemorrhage (red arrows).

A 7‐year‐old boy presented to King Khaled Eye Specialist Hospital and Research Center with decreased vision in his left eye for 2 months. His medical, surgical, trauma, systemic, and family histories were unremarkable. Best‐corrected visual acuity (BCVA) was 20/25 in the right eye and 20/100 in the left eye. Anterior segment examination of both eyes was within normal limits (WNLs). Dilated fundus examination (DFE) was unremarkable in the right eye. In the left eye, a whitish, central macular subretinal lesion measuring approximately half a disk diameter was noted, surrounded by a dehemoglobinized subretinal hemorrhage (Figure 1A). Optical coherence tomography (OCT) confirmed the presence of subretinal hyporeflectivity and hyperreflectivity, representing both the subretinal fluid and hemorrhage, respectively (Figure 2A,B). OCT angiography (OCT‐A) demonstrated clear evidence of an active CNV with intralesional blood flow (Figure 2C). The B‐scan ultrasonography revealed ocular wall calcification with posterior acoustic shadowing in the macular region (Figure 3A–C). The right eye appeared WNL on all aforementioned imaging modalities. Routine laboratory investigations, including complete blood count, glucose‐6‐phosphate dehydrogenase testing, and sickle cell screening, were unremarkable. Based on the clinical and imaging findings, we diagnosed CO complicated by secondary CNV. The patient was scheduled to receive an intravitreal aflibercept injection in the left eye under general anesthesia. One month after the first injection, BCVA improved to 20/40 in the left eye. Imaging demonstrated reduced subretinal blood flow compared with that at the first visit (Figures 1B and 4). OCT demonstrated less subretinal fluid and hemorrhage in the left eye (Figure 2D,E). A second aflibercept injection was administered per the management plan.

**Figure 5:**
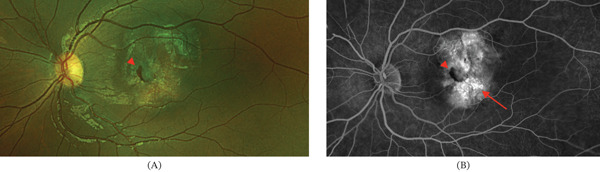
Ultrawide pseudocolor fundus and fluorescein angiography (FA) images of the left eye taken at the 4‐month follow‐up. (A) Fundus image showing a new subretinal hemorrhage. (B) Late arteriovenous phase FA showing the diffuse macular subretinal hyperfluorescence staining of fibrotic tissue (large red arrow) with central hypofluorescence from the blocking effect of the subretinal hemorrhage (arrowhead).

**Figure 6:**
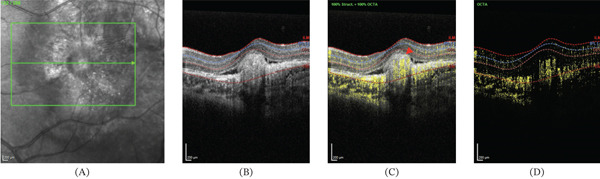
Macular OCT and OCT‐A of the left macula at the 4‐month follow‐up visit. (A, B) OCT‐A retinal layer segmentation showing subretinal fluid. (C) A blood flow layer was added, showing active flow (red arrowhead). (D) Without the anatomical layer.

**Figure 7:**
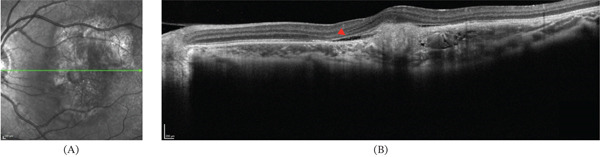
(A,B) Macular OCT of the left eye macula at the 5‐month follow‐up visit, 1 month after the third intravitreal aflibercept injection, showing improved subretinal fluid (red arrow).

At the 3‐month follow‐up, DFE of the left eye showed no signs of CNV activity (Figure 1C). OCT revealed the complete resolution of subretinal fluid and hemorrhage, with thickened choroidal areas and hyperreflective dots (Figure 2F,G). Despite these anatomical improvements, BCVA declined to 20/100 in the left eye. At the 4‐month follow‐up, BCVA further decreased to 20/160, with evidence of CNV reactivation and a new subretinal hemorrhage, as confirmed via fluorescein angiography (Figure 5A,B), OCT, and OCT‐A (Figure 6A–D). A third intravitreal aflibercept injection was administered. One month later, BCVA partially improved to 20/80, with improved subretinal hemorrhage on clinical examination and OCT imaging (Figure 7A,B).

**Figure 8:**
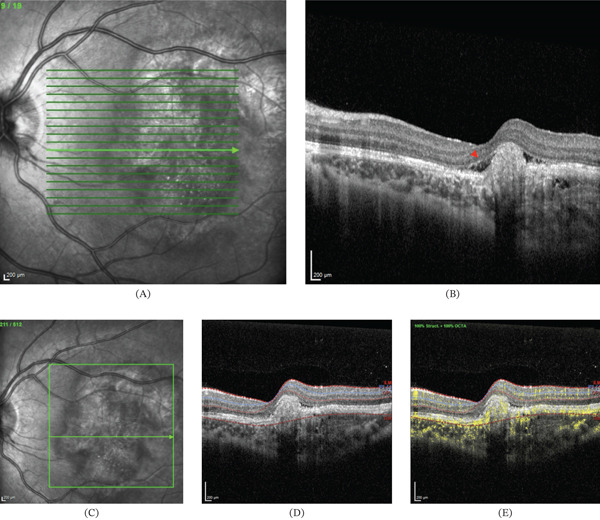
Macular OCT and OCT‐A of the left macula at the 6‐month follow‐up. (A,B) Structural OCT showing recurrent subretinal fluid. (C–E) OCT‐A of the same macula, showing the corresponding B‐scan with automated retinal layer segmentation and active intralesional flow within the choroidal neovascular membrane. The green arrows are device‐generated markers denoting the location and direction of the corresponding B‐scan.

At the 6‐month follow‐up, BCVA declined to 20/125, with recurrent subretinal fluid evident on OCT and OCT‐A (Figure 8A–E), and a fourth intravitreal aflibercept injection subsequently administered.

At 9 months of follow‐up, the patient presented with no new complaints. Visual acuity was 20/100 in the left eye after four aflibercept injections. DFE and the OCT images confirmed an inactive CNV, and the patient was followed up for 2 months for monitoring and administering aflibercept injections if any CNV activity was noted. Table 1 summarizes the changes in BCVA and imaging findings during the follow‐up.

**Table 1:** Timeline of visual acuity and imaging findings in relation to aflibercept injections.

Timeline	BCVA	Imaging findings	OCT and OCT‐A status	Aflibercept injection administered
Baseline	20/100	Subfoveal choroidal osteoma with subretinal hemorrhage	Active CNV with SRF	Yes
1 month	20/40	Reduced subretinal hemorrhage	Active CNV with less SRF	Yes
3 months	20/100	No clinical hemorrhage	Inactive CNV	No
4 months	20/160	New subretinal hemorrhage	Reactivated CNV	Yes
5 months	20/80	—	SRF resolution	No
6 months	20/125	Recurrent SRF	Active CNV	Yes
9 months	20/100	Stable fundus appearance	Inactive CNV	No

Abbreviations: BCVA, best‐corrected visual acuity; CNV, choroidal neovascularization; OCT, optical coherence tomography; OCT‐A, OCT angiography; SRF, subretinal fluid.

## 3. Discussion

Several aspects of CO remain poorly understood, including its etiology, risk factors, prevalence, and pathophysiology, largely due to its rarity [2]. Ketkar et al. published the largest retrospective study to date in 2025, including 80 patients over 12 years in India [7]. Previously, the largest series was reported by Shields et al., comprising 61 patients followed over 26 years [3]. Additionally, the Phase III MINERVA study, which evaluated ranibizumab for CNV secondary to uncommon causes and supported its approval for this indication, included 29 patients with miscellaneous CNV etiologies [8]. However, none of the included cases was secondary to CO, underscoring the rarity of CNV associated with this condition.

In the absence of standardized diagnostic and management guidelines, treatment is primarily aimed at controlling complications of CO, particularly CNV [2, 5]. Variable treatment modalities have been proposed, including surgical excision, argon and krypton laser photocoagulation, photodynamic therapy, and transpupillary thermotherapy. However, these approaches have shown limited success and may cause retinal damage due to their invasive nature, potentially worsening visual acuity [2]. More recently, intravitreal anti‐VEGF agents, such as ranibizumab and bevacizumab, have emerged as less invasive treatment options. Whereas many studies report favorable short‐term outcomes, others indicate limited long‐term benefit without regular follow‐up, given the high recurrence rates of CNV [5, 9–11].

The use of aflibercept in pediatric patients with CNV secondary to CO is extremely limited in the literature. Khan et al. reported a case of a 13‐year‐old girl with CNV secondary to CO who initially showed improvements after bevacizumab and ranibizumab injections [12]. However, because these agents were unavailable later, treatment was switched to aflibercept. Although visual acuity remained stable, OCT demonstrated increased subretinal fluid, prompting a switch back to bevacizumab. In contrast, the present patient was younger and was treated primarily with aflibercept. Similar to the case reported by Khan et al., recurrent CNV activity occurred and required repeated injections. In adults, published reports of aflibercept use have shown variable outcomes, with some cases demonstrating marked visual improvement, whereas others showed only short‐lived improvement followed by recurrent activity and visual decline [13].

Aflibercept is a newer FDA‐approved intravitreal anti‐VEGF agent that has become routinely used for retinal vascular and neovascular conditions at our center and was selected as the primary intervention in the present case. The patient was treated using a pro re nata regimen, with retreatment guided by persistent or recurrent CNV activity on clinical examination and multimodal imaging. Although no head‐to‐head studies have compared aflibercept with other anti‐VEGF agents for CO‐related CNV, large studies in neovascular age‐related macular degeneration (AMD) suggest that aflibercept provides visual outcomes comparable to ranibizumab [14]. Additionally, switching to aflibercept improved anatomical outcomes in some eyes with neovascular AMD resistant to bevacizumab or ranibizumab [15, 16]. In the present case, the use of aflibercept resulted in short‐term anatomical and transient functional improvement, although recurrence was observed, requiring repeated injections. Larger studies with longer follow‐up are required to better define anti‐VEGF therapy′s long‐term efficacy in CO.

Early detection of CO is critical for preserving long‐term visual acuity. Characteristic features, such as branching, spider‐like tumor vessels, and well‐demarcated geographic borders, can aid early diagnosis [17]. Tumor color may also indicate disease stage, with early lesions appearing orange‐red, and later stages showing a yellowish hue due to retinal pigment epithelium thinning.

Several differential diagnoses may be considered for a calcified‐appearing choroidal lesion in a child, including amelanotic choroidal melanoma, retinoblastoma, granuloma, and other amelanotic choroidal tumors [2]. In this case, the diagnosis of CO was considered based on the well‐demarcated whitish macular lesion and B‐scan ultrasonography showing ocular wall calcification with posterior acoustic shadowing [2]. The presence of active CNV further supported the diagnosis, although CNV is not a typical presenting feature of several of these alternative diagnoses. OCT‐A demonstrated abnormal intralesional flow consistent with active CNV on the avascular complex slab. Given the potential limitations of OCT‐A in elevated lesions, including retinal distortion and segmentation artifacts, OCT‐A findings were interpreted in conjunction with clinical and imaging findings. Automated segmentation was used, and no manual segmentation correction was performed.

The decline in BCVA at the 3‐month follow‐up, despite apparent anatomical improvement on OCT, may be explained by the multifactorial nature of visual loss in the present case. Although subretinal fluid and hemorrhage had resolved, visual recovery may have been limited by the subfoveal location of the lesion. Visual acuity in CO is influenced by several factors, including tumor location, particularly subfoveal macular involvement, and the development of CNV, which is associated with subretinal fluid accumulation and/or decalcification [2, 3, 17]. These changes may lead to photoreceptor degeneration and irreversible visual dysfunction. Therefore, anatomical improvement after anti‐VEGF therapy does not necessarily mean functional improvement, particularly when photoreceptor damage or subfoveal scarring is present. Aylward et al. reported a 58% risk of legal blindness (≤ 20/200) within 10 years, increasing to 62% by 20 years [18].

## 4. Conclusion

Although CO is more commonly reported in adult females, this case demonstrates that it should also be considered in young male children. Early treatment with aflibercept was associated with short‐term anatomical and transient functional improvement in managing CNV secondary to CO in a pediatric patient. However, the visual response was unstable, and disease recurrence was observed, underscoring the importance of regular follow‐up visits to preserve long‐term visual acuity. Given the rarity of CO, further studies with larger sample sizes and extended follow‐up periods are necessary to evaluate the long‐term effectiveness of anti‐VEGF therapy and establish evidence‐based treatment guidelines.

## Author Contributions

Abdullah Altalhi: conceptualization, investigation, data curation, visualization, project administration, writing—original draft, and writing—review and editing; Anas Alqurashi: investigation, data curation, visualization, writing—original draft, and writing—review and editing; Mohammed Albassam: investigation, resources, data curation, validation, and writing—review and editing; Abdulmalik Alyahya: conceptualization, methodology, resources, supervision, validation, and writing—review and editing.

## Funding

No funding was received for this manuscript.

## Disclosure

All authors have read and approved the final version of the manuscript and agreed to be accountable for all aspects of the work.

## Ethics Statement

This study was approved by the Institutional Review Board of the King Khaled Eye Specialist Hospital and Research Center (RP 25178‐CR), and written informed consent was obtained from the patient′s guardian for publication of this case report and accompanying images. Patient information was sufficiently anonymized according to the ICMJE guidelines.

## Conflicts of Interest

The authors declare no conflicts of interest.

## Data Availability

Data are available upon reasonable request.
